# The effects of short-term fasting on quality of life and tolerance to chemotherapy in patients with breast and ovarian cancer: a randomized cross-over pilot study

**DOI:** 10.1186/s12885-018-4353-2

**Published:** 2018-04-27

**Authors:** Stephan P. Bauersfeld, Christian S. Kessler, Manfred Wischnewsky, Annette Jaensch, Nico Steckhan, Rainer Stange, Barbara Kunz, Barbara Brückner, Jalid Sehouli, Andreas Michalsen

**Affiliations:** 1 Institute of Social Medicine, Epidemiology and Health Economics, Charité – Universitätsmedizin Berlin, corporate member of Freie Universität Berlin, Humboldt-Universität zu Berlin, and Berlin Institute of Health, Berlin, Germany; 2 Department of Internal and Integrative Medicine, Immanuel Krankenhaus Berlin, Berlin, Germany; 3 Department of Mathematics and Computer Science, University of Bremen, PB 330440, Bremen, 28334 Germany; 4 Department of Gynecology, Krankenhaus Waldfriede, Argentinische Allee 40, 14163, Berlin, Germany; 5 Department of Gynecology, Charité - Universitätsmedizin Berlin, Berlin, Germany

**Keywords:** Breast cancer, Chemotherapy, Fasting, Pilot study, Quality of life, Ovarian cancer

## Abstract

**Background:**

This pilot trial aimed to study the feasibility and effects on quality of life (QOL) and well-being of short-term fasting (STF) during chemotherapy in patients with gynecological cancer.

**Methods:**

In an individually-randomized cross-over trial patients with gynecological cancer, 4 to 6 planned chemotherapy cycles were included. Thirty-four patients were randomized to STF in the first half of chemotherapies followed by normocaloric diet (group A;*n* = 18) or vice versa (group B;*n* = 16). Fasting started 36 h before and ended 24 h after chemotherapy (60 h-fasting period). QOL was assessed by the FACIT-measurement system.

**Results:**

The chemotherapy-induced reduction of QOL was less than the Minimally Important Difference (MID; FACT-G = 5) with STF but greater than the MID for non-fasted periods. The mean chemotherapy-induced deterioration of total FACIT-F was 10.4 ± 5.3 for fasted and 27.0 ± 6.3 for non-fasted cycles in group A and 14.1 ± 5.6 for non-fasted and 11.0 ± 5.6 for fasted cycles in group B. There were no serious adverse effects.

**Conclusion:**

STF during chemotherapy is well tolerated and appears to improve QOL and fatigue during chemotherapy. Larger studies should prove the effect of STF as an adjunct to chemotherapy.

**Trial registration:**

This trial was registered at clinicaltrials.gov: NCT01954836.

## Background

Experimentally, short-term fasting (STF) protects healthy cells against the adverse effects of chemotherapy while making tumor cells more vulnerable to it [1]. An increasing body of evidence from basic research points to beneficial effects of intermittent and periodic fasting in chronic disease [2–7]. Fasting promotes pronounced changes in metabolic pathways and cellular processes such as stress response (hormesis), autophagy and decreases IGF-1 that affects other factors as Akt, Ras and mammalian target of rapamycin (mTOR) to downregulate cell growth and proliferation [8]. In primates and in rodents caloric restriction and intermittent fasting are associated with reduced cancer risk [9, 10]. Chemotherapy is a mainstay in the treatment of malignant tumors, but frequently limited by its toxicity. Recently it has been shown, that the effects of fasting on susceptibility to chemotherapy differ between normal cells and cancer cells, a phenomenon described as differential stress resistance (DSR) [11, 12]. Experimental data indicate that fasting states may promote the protection of normal cells, but not cancer cells during chemotherapy, as oncogenic pathways inhibit the stress resistance. In healthy cells pronounced metabolic and gene expression changes are induced by fasting, including upregulation of DNA repair pathways and antioxidants, partly mediated by the shut down of pathways such as IGF-1/Akt and mTOR [13].

As weight loss may negatively affect the prognosis of cancer patients with cancer, short- term fasting (STF), which is not related to long-term weight loss and its adverse effects has been introduced in basic research as a potential add-on treatment during chemotherapy [14]. So far, experimental data consistently show that the combination of short-term fasting cycles with chemotherapy is effective in enhancing chemotherapeutic tolerability and efficacy and thus has high translational potential [1]. In a first case series on 10 patients with various types of cancer STF was found to be feasible and reduced severity of chemotherapy-induced side effects. [8]. In a recent randomized pilot-study 13 women with HER2-negative breast cancer receiving neo-adjuvant chemotherapy were randomized to STF during chemotherapy or to eat a common healthy diet. Fasting reduced the hematological toxicity of chemotherapy and induced a faster recovery of DNA damage [12]. The present pilot study was designed to assess the effect of a 60 h-STF on quality of life (QOL) in patients with gynecological cancer under chemotherapy. Based on the experimental evidence we hypothesized that fasting increases QOL and reduces fatigue during chemotherapy compared to standard nutrition [15]. By means of an explorative cross-over design we compared QOL, general well-being and fatigue across all fasted chemotherapy cycles versus all chemotherapy cycles with normocaloric diet.

## Methods

This study was designed as a randomized, individually controlled cross-over trial. The study design was chosen on the background of the anticipated heterogeneity of the study population and chemotherapy protocols. All study participants gave their written informed consent. The study protocol was reviewed and approved by the Ethics Committee of the Charité-University Medical Center, Berlin (EA4/088/13). Patients were enrolled between November 2013 and March 2015; interventions and follow-up were completed by August, 2015. Study procedures and data collection were carried out at the outpatient department of the Charité -University Berlin at Immanuel Krankenhaus Berlin.

### Study procedures

Patients were referred by three gynecological hospital departments, two centers for breast cancer care and the Charité European Center for ovarian cancer. Potential participants were screened for eligibility during an appointment at the study center, and eligible candidates were scheduled for an enrollment appointment. Each eligible participant was randomly assigned to two different sequences of nutrition during the scheduled chemotherapy. Group A was randomized to a STF of 60 h during the first three of scheduled 6 chemotherapies (36 h before to 24 h after the chemotherapy) followed by normocaloric nutrition during the following 3 chemotherapies. Group B was allocated to a vice versa sequence of nutrition. Between the chemotherapy cycles all patients were advised to follow their common diet. All patients received an approximate 1 h counselling by dieticians experienced in fasting treatments.

All measurements were performed baseline and 8 days after each chemotherapy cycle. Subjects height/body weight were measured following a standardized protocol. For assessment of QOL, general well-being and fatigue validated inventories were used. Adverse effects were assessed by two 2 interviews during and at the end of fasting and by means of a diary.

### Patients

All women had a confirmed diagnosis of breast cancer or ovarian cancer and a scheduled chemotherapy. Eligibility criteria included age ≥ 18 years; BMI ≥ 19 kg/m^2^; WHO performance status 0–2; anticipated life expectancy of > 3 months; Exclusion criteria included: Type-1 diabetes or intensified insulin treatment; myocardial infarction, stroke or pulmonary embolism within the last 3 months; unstable heart disease; renal failure, history of eating disorder; dementia, psychosis, impaired physical mobility.

**Fig. 1:**
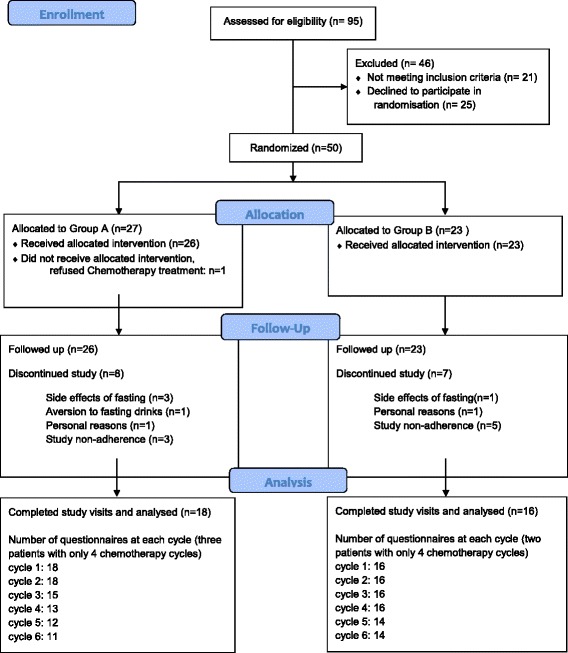
Study flow chart

One hundred twenty-one patients were screened, 50 patients fulfilled the inclusion criteria and were recruited to the study. The patient flow chart is given in Fig. 1. One patient cancelled the complete chemotherapy treatments initially. Although STF was generally well tolerated, four patients withdrew from STF after having experienced minor side effects of STF like headache (two cases), hyperventilation during first chemotherapy (one case) and general subjective weakness (one case). One patient reported an aversion to fasting nutrients. Thus, in total 5 patients (10%) dropped out related to the fasting intervention. Two patients withdrew from the study because of personal reasons (family problems), each in the diet phase. Eight subjects could not be assessed for the follow-ups as a result of non-adherence with the study because of time restrictions and complete unwillingness to fill out further study documents (three in the fasting phases, five in the diet phases). These drop-outs were not related to any adverse effects of the study interventions as assessed by telephone interviews. A total of 34 patients with primary BC (*n* = 29), advanced BC (*n* = 1) and OC (*n* = 4) were analysed.

### Interventions

All patients received standard oncological care as determined by their individual requirements. Patients in both groups were advised to maintain their regular physical activity and to abstain from other new integrative or supportive treatments during the study period.

### Chemotherapeutic drugs and standard therapy

Chemotherapeutic drugs used in this study were taxanes (docetaxel (D), paclitaxel (T)), platinum agents (carboplatin (P), cyclophosphamide (C), anthracyclines (epirubicin (E), doxorubicin (A), methotrexatate (M), fluorouracil (F), the IgG1 antibody bevacizumab (Avastin) and for patients with HER2/neu overexpression pertuzumab or trastuzumab. These were given in various standard combinations according to guideline-based treatment protocols. For patients with breast cancer we had the following regimens: EC, FEC-D; FEC-D + trastuzumab, AC-T,EC-T,TAC and D+ pertuzumab+ trastuzumab. For patients with ovarian cancer we used: P mono, P + T, EC-T + P and P + T + bevacizumab. Standard antiemetics and medication to prevent hypersensitivity reactions were administered, including dexamethasone and 5HT3 inhibitors.

### Fasting intervention

All patients received individual dietary counselling according to their individual needs in order to correctly perform the fasting program and the normocaloric diet. The fasting program was adapted to the established approach of modified fasting used in our hospital for many years and evaluated in studies in patients with rheumatic diseases and chronic pain [16]. The fasting period started 36 h before chemotherapy and ended 24 h after the end of the chemotherapy resulting in a total fasting period of 60 h. During the fasting period subjects received unrestricted amounts of water, herbal tea, 2x100cl vegetable juice and small standardized quantities of light vegetable broth with a maximum total daily energy intake of 350 kcal. Compliance with the fasting regimen was assessed by telephone calls and personal interviews at the end of the chemotherapy cycles.

### Standard nutrition period

All patients were advised to follow a normocaloric Mediterranean diet throughout this study phase including the chemotherapy days.

### Randomization

Patients were randomly allocated to treatment groups (block-randomization with randomly varying block sizes (SAS/Base® statistical software (SAS/Base® statistical software)). For each patient sealed, sequentially numbered envelopes containing treatment assignments were prepared. Allocation of treatment was not blinded.

### Inventories for outcome assessment

For measuring health-related QOL we used the functional assessment of chronic illness therapy (FACIT©) measurement system. The Functional Assessment of Cancer Therapy-General (FACT-G©) forms the generic core questionnaire of all FACIT© scales. The FACIT© scales are constructed to complement the FACT-G© scale by addressing relevant disease-, treatment-, or condition-related issues not already covered in the general questionnaire. The Trial Outcome Index (TOI) is a measure of physical aspects of QOL. It is the sum of the FACT-G subscales of physical well-being (PWB), functional well-being (FWB), and any FACIT© disease-, treatment-, or condition-specific scale. For additional concerns we used the FACIT-F©, a 13-item questionnaire that assesses self-reported fatigue and its impact upon daily activities. Altogether we obtained 8 different scales and subscale scores: the subscales PWB, EWB, SWB, FWB as compounds of the FACT-G© scale (27 items); the additional subscale FACIT-F© (13 items); the TOI-FACIT-F© (27 items) and the total FACIT-F© scale as union of the FACT-G© and FACIT-F© scales (40 items).

### Statistical analysis

This randomized cross-over pilot study aimed to give first insight into QOL and tolerability to chemotherapy in breast or ovarian cancer patients with STF versus normocaloric diet during chemotherapy. As a pilot study it aimed to enable the design of a further confirmatory trial that is anticipated.

All FACIT© scales were scored with a higher score indicating better well-being. Here, we reversed response scores on negatively phrased questions, then added the item responses. The scores were obtained in accordance with the formula that had been previously established by the FACIT© system. In cases where individual questions were skipped, scores were prorated using the average of the other answers in the subscale (prorated subscale score = [sum of the item scores]*[N of items in subscale]/[N of items answered]) as long as more than 50% of the items were answered (minimum of 4 items for the subscales). The FACT-G score was considered appropriate as long as at least 22 of 27 FACT-G items were completed (≥80%). Inter-subscale correlations were computed using Pearson correlation, and the reliability of the internal consistency for all scales were assessed by computing Cronbach’s alpha. When Cronbach’s alpha exceeded 0.90, the scale was considered to have sufficient precision for individual classification or diagnosis. MIDs of fasting and non-fasting groups were used to find clinically meaningful improvements in symptoms and to aid the interpretation of group differences and changes in QOL over time. A MID for scores of scales is defined by the “smallest change in a score in the domain of interest that patients perceive as important, either beneficial or harmful, and that would lead the clinician to consider a change in the patient’s management” [17]. MID values over 3–7 (mean 5) for FACT-G© and over 3–4 for Fatigue subscale and 6 for Total FACT-F were considered significant.

The sample size was calculated for the group A starting with STF assuming an equivalence margin of 5 i.e. the MID of FACT-G, a true difference of 7 and a standard deviation of 3. A sample size of 16 achieves a power of 82% with a significance level of 0.05. Assuming the same condition for the group B we obtain 32 patients as minimum.

Continuous variables with a normal distribution were expressed as mean value and standard deviation. Normality was tested with the Shapiro–Wilks test. Statistical comparison of baseline characteristics and outcomes was performed using the × 2 test with Yates correction or the Fisher exact test, when appropriate, for categorical variables. For continuous variables we used the two-tailed Student’s t-test respectively the independent-samples Mann-Whitney U test in order to test the null hypothesis that the distribution of a variable is the same across the groups A and B.

Calculations were performed with NCSS (Version 10), R (version 3.1.0) and IBM SPSS Statistics for Windows (Version 22.0. Armonk, NY: IBM Corp).

## Results

The median age of the 34 patients was 51y (range 28-69y). Eighteen patients started with fasting during the first half of chemotherapy cycles (group A) whereas the other 16 patients (group B) started with normal diet (Table 1).

**Table 1:** Legend: Baseline characteristics for both groups. Group A: Fasting for the first half of cycles of chemotherapy and eating a normocaloric diet for the second half of cycles; Group B: Eating a normocaloric diet for the first half of cycles of chemotherapy and fasting for the second half of cycles

Basic characteristics		Total 34	Group A 18 (52.9%)	Group B 16 (47.1%)	Significance
Age at diagnosis		mean: 51.6 (SD 8.4) (median:51) Range: 28–69	mean: 49.8 (SD 9.1) (median:51) (Range: 28–63	mean: 53.6 (SD 7.3) (median: 52) Range:44–69	0.195
Menopausal status	premenopausal	24	13 (72.2)	11 (68.7)	0.824
	postmenopausal	10	5 (27.8)	5 (31.3)	
Tumor entity	primary breast cancer	25 (73.5)	12 (66.7)	13 (81.3)	0.691
	advanced breast cancer	5 (14.7)	3 (16.7)	2 (12.5)	
	ovarian cancer	3 (8.8)	2 (11.1)	1 (6.3)	
	advanced ovarian cancer	1 (2.9)	1 (5.6)	0 (0.0)	
T-categories	T0	1 (2.9)	0 (0.0)	1 (6.3)	0.323
	T1	14 (41.2)	7 (38.9)	7 (43.8)	
	T2	13 (38.2)	9 (50.0)	4 (25.0)	
	T3	6 (17.6)	2 (11.1)	4 (25.0)	
Nodal status	pNx	1 (2.9)	1 (5.6)	0 (0.0)	0.434
	pN0	16 (47.1)	7 (38.9)	9 (56.3)	
	pN1	17 (50.0)	10 (55.6)	7 (43.8)	
Grading	G1	1 (2.9)	1 (5.6)	0 (0.0)	0.298
	G2	17 (50.0)	7 (38.9)	10 (62.5)	
	G3	16 (47.1)	10 (55.6)	6 (37.5)	
Breast cancer intrinsic subtypes (*n* = 30)	Luminal A	3 (10.0)	3 (20.0)	0 (0.0)	0.098
	Luminal B / HER2-	11 (36.7)	4 (26.7)	7 (46.7)	
	Luminal B / HER2+	4 (13.3)	2 (13.3)	2 (13.3)	
	triple negative	9 (30.0)	6 (40.0)	3 (20.0)	
	HER2 overexpressing	3 (10.0)	0 (0.0)	3 (20.0)	
Therapy (breast cancer)	CT	25 (83.3)	13 (86.7)	12 (80.0)	0.624
	CT + Anti-HER2	5 (16.7)	2 (13.3)	3 (20.0)	
Therapy (ovarian cancer)	CT	3 (75.0)	3 (100.0)	0 (0.0)	0.046
	CT + Anti-HER2 + Anti-VEGF	1 (25.0)	0 (0.0)	1 (100.0)	
Body Mass Index	normal (BMI ≤25)	19 (55.9)	9 (50.0)	10 (62.5)	0.364
	overweight (BMI: 25–30)	13 (38.2)	7 (38.9)	6 (37.5)	
	obese (BMI > 30)	2 (5.9)	2 (11.1)	0 (0.0)	

Fasting was safe and all reported side effects were of low grade and at a level that did not interfere with daily activities. Minor adverse effects during all cycles, and mainly during the first STF cycle, included headache (5×), hunger (5×), slight nausea after intake of broth or juices (11×) and one orthostatic reaction. There was one wound infection, unrelated to fasting. Regarding tolerability of chemotherapy there were no common toxicity criteria grade III/IV adverse events documented.

### Changes in body weight/body mass index

Weight gain is a common problem for breast/ovarian cancer patients treated with chemotherapy and in particular with anthracyclines+taxanes [18]. In this trial there were no significant (*p* > 0.3) changes in weight. Mean body weight (BMI) of the patients in group A was 73 kg (26.1) at the beginning and 72.3 kg (25.8) at the end of the trial. For group B the corresponding values are 67.9 kg (23.7) and 68.5 kg (24.2).

Throughout all chemotherapy cycles of the 34 patients we documented 102 cycles fasted and 74 cycles on normocaloric diet. The numerical difference between fasted and non-fasted cycles is a result of 5 patients who didn’t want to switch to normocaloric diet after having fasted the first 3 cycles. Compliance with the fasting protocol, as checked by diaries, telephone calls and interviews appeared good.

### Baseline

The means of the various scales and subscales were worse for patients with ovarian cancer compared to patients with breast cancer at baseline (day 0, cycle 1) (OC vs BC: FACT-G 66.8 (±21.7) vs 80.5 (±15.3); FACIT-F 33.5 (±10.3) vs 39.7 (±10.2); Total FACIT-F 100.3(±31.8) vs 120.2 (±21.7)). The internal consistencies were excellent for the various questionnaires: (Cronbach’s alpha: α > 0.9). FACT-G (α = 0.91), FACIT-F (α = 0.96), TOI FACIT-F (α = 0.97) and total FACIT-F (α = 0.96). There were statistically no significant differences in FACT-G respectively its subscales between both groups at baseline except for social/family well-being *p* = 0.042).

### Chemotherapy courses

We used a 2 × 2 crossover design AB|BA (A = SFT; B = normocaloric diet) with cross-over at cycle 4. Five patients had a total of only four cycles of chemotherapy, so we aligned the first two cycles to c1 and c2 and the second two cycles to c4 and c5.

**Fig. 2:**
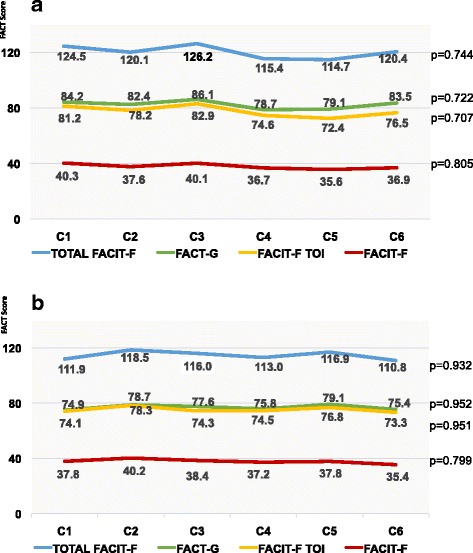
**a**: Pretest values at cycles (C1-C6) in FACIT-F(FS), FACIT-F, FACIT-F TOI, FACT-G, and Total FACIT-F, Group A, Day 0. **b**: Pretest values at cycles (C1-C6) FACIT-F(FS), FACIT-F, FACIT-F TOI, FACT-G, and Total FACIT-F Group B, Day 0

The mean values of all scales at day 0 (before chemotherapy) were not significantly different for group A and B across the 6 cycles, i.e. the patients recovered within 3 weeks from each cycle of chemotherapy with respect to QOL (Fig. 2a/b) Hence, there seemed to be no carryover effects from SFT respectively normocaloric diet during the first three cycles to the second period.

### Change of QOL after chemotherapy

Within group A we found a statistically and clinically significant beneficial effect of STF during chemotherapy (cycles c1-c3) versus normocaloric diet (cycles c4-c6) on QOL and fatigue. In group B fasted cycles (c4-c6) were not associated with a significant reduction of chemotherapy-induced QOL and fatigue compared to regular diet (c1-c3) (Table 2).

**Table 2:** Mean and standard deviations for outcome parameters with mean group differences and 95%CI for FACT-G, FACIT-F, TOI FACIT-F, and Total FACIT-F between cycles with fasting and regular diet of group A and B at day 8 after chemotherapy

Descriptives								
Group	Cycle	N	Mean	Std. Deviation	95% Confidence Interval for Mean		Mean difference	Statistical significance
					Lower Bound	Upper Bound		
FACT-G Group A	first half of a 4–6 cycle	52	71.1	18.7	65.9	76.3	9.2	0.041
	second half of a 4–6 cycle	27	61.9	18.5	54.6	69.2		
FACT-G Group B	first half of a 4–6 cycle	46	76.0	17.2	70.9	81.1	2	0.576
	second half ofa 4–6 cycle	47	74.0	17.6	68.8	79.2		
FACIT-F (FS) Group A	first half of a 4–6 cycle	52	33.9	13.4	30.2	37.6	9.1	0.006
	second half of a 4–6 cycle	27	24.8	13.7	19.4	30.2		
FACIT-F (FS) Group B	first half of a 4–6 cycle	46	33.4	14.0	29.3	37.6	1.8	0.521
	second half of a 4–6 cycle	47	31.7	12.6	28.0	35.4		
FACIT-F TOI Group A	first half of a 4–6 cycle	52	66	25	59	72.9	16.2	0.009
	second half of a 4–6 cycle	27	49.8	26.4	39.4	60.3		
FACIT-F TOI Group B	first half of a 4–6 cycle	46	68.0	25.2	60.5	75.5	4.2	0.41
	second half of a 4–6 cycle	47	63.8	23.9	56.8	70.8		
FACIT-F Total Group A	first half of a 4–6 cycle	52	105	30.4	96.6	113.5	18.3	0.013
	second half of a 4–6 cycle	27	105	30.5	74.6	98.8		
FACIT-F Total Group B	first half of a 4–6 cycle	46	109.5	29.8	100.6	118.3	4.2	0.531
	second half of a 4–6 cycle	47	105.7	28.3	97.3	114.0		

**Fig. 3:**
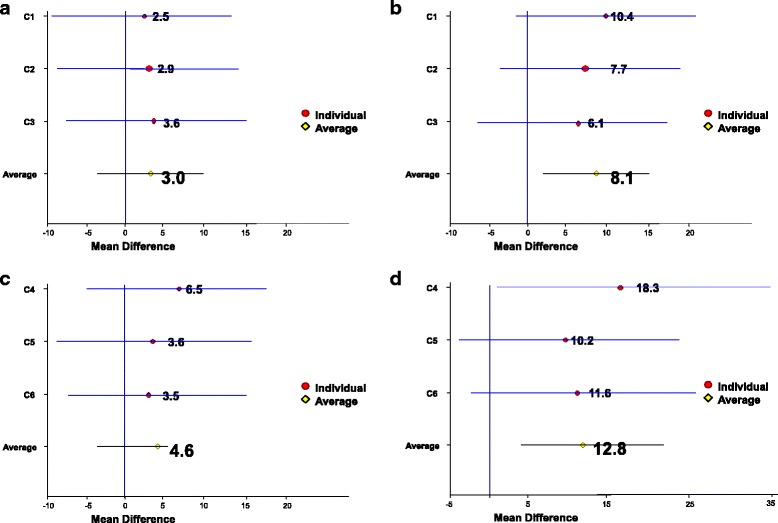
FACT-G Forest plot of mean difference. **a**) Group A (pretest – day 8, cycles c1-c3 = fasting). **b**) Group B (pretest – day 8 (cycles c1-c3 = normocaloric). **c**) Group B (pretest(c4) – day 8, cycles c4-c6 = fasting) and d) Group A (pretest(c4) – day 8, cycles c4-c6 = normocaloric)

When analysing changes of QOL within the fasted or non-fasted periods by applying minimally important differences (MID for FACT-G = 5) we found for patients in group A within the first three fasted cycles a decrease of FACT-G (mean = 3.0) that was less than the MID. Thus, patients under STF did not perceive the change between FACT-G at day 0 and day 8 after chemotherapy as important. In contrast, the change of QOL for patients of group A across the normocaloric diet cycles c4-c6 was greater than the MID (mean 12.8.), these patients perceived the chemotherapy-induced reduction of QOL as important [Fig. 3a,d].

Accordingly, the mean differences (day0 – day 8) of TOI FACIT-F of group A was 10.5 for first three cycles and 25.1 for cycles 4–6**.** Patients who fasted within the first three cycles had a clinically significantly lower difference of TOI FACIT-F compared to the same patients during cycles 4–6 on a regular diet.

The mean differences of TOI FACIT-F of group B was 13.1 for first three cycles and 10.8 for cycles 4–6**.** Patients who fasted within the first three cycles had a clinically significantly lower difference of TOI FACIT-F compared to the same patients during cycles 4–6 on a regular diet. There was no significant difference between the mean differences of TOI FACIT-F for group A and group B during the fasting cycles (10.5 vs 10.8).

In group B we found no statistically significant differences between the first 3 cycles and the second three cycles. However, when analysing changes of QOL within the fasted or non-fasted periods by applying MID we found an average of mean difference of FACT-G for the cycles with STF of 4.6 and with normocaloric diet of 8.1 [Fig. 3b, c]. Thus, again patients with normocaloric diet had a mean difference above the MID in that phase whereas with STF the mean difference was 4.6 i.e. below the MID. Fig. 3a-d summarizes the forest plots of the chemotherapy-induced changes of FACT-G in each of the two dietary phases in both groups.

In general, STF was very well accepted by the patients. At the final consultation the majority of patients reported better tolerance to chemotherapy with STF, the patient general assessment of the effectiveness of STF revealed “good” or “very good” for 28 patients, “moderate” in 5 patient s and “no effect” in 1 patient. Thirty-one patients declared that they would fast again during chemotherapy, 3 patients declared that they would not like to fast again during chemotherapy.

## Discussion

This is the first clinical study to explore the effects of STF on QOL, fatigue and wellbeing during chemotherapy. Experimentally, STF has been documented to induce profound changes in gene expression and cellular metabolism that render normal cells more resistant to oxidative stress and thus may confer benefit in the situation of cancer treatment by chemotherapy [11].

The present study included women with breast cancer and ovarian cancer and used an intra-individual randomized cross-over study design to balance for the heterogeneity in disease states and chemotherapy protocols. STF with a period of 60 h did not induce weight loss and was associated with only minor adverse effects that were rated as not meaningful by the patients and did not interfere with daily activities.

In general, STF led to a better tolerance to chemotherapy with less compromised QOL and reduced fatigue within the 8 days after chemotherapy.

To date, there are only three previous reports in smaller samples that evaluated the effects of STF during chemotherapy. In a first case series Safdie et al. described 10 cases in which patients with various malignancies had voluntarily fasted prior to and after chemotherapy with various lengths of the fasting periods [8]. Six patients that underwent chemotherapies with and without fasting reported a reduction in fatigue, weakness, and some gastrointestinal side effects by fasting.

In a recent pilot study De Groot et al. investigated a 48 h zero-calorie fasting during TAC chemotherapy in 13 women with confirmed HER-2-negative stage II and III breast cancer. Beside metabolic, endocrine and hematologic parameters, DNA damage in PBMCs was also assessed and side effects were evaluated according to the Common terminology Criteria for Adverse events (CTCAE). Two patients withdrew from fasting after the third chemotherapy because of clinical deterioration not related to fasting. Fasting was safe and had beneficial effects on hematologic toxicity and possibly on DNA damage in healthy cells (lymphocytes and myeloid cells) [19].

In the third report, a recent uncontrolled dose- escalation and feasibility study, Dorff et al. investigated 20 patients with three different fasting periods (24, 48 and 72 h). Sixteen of the patients were compliant with the fasting regimen (< 200 kcal/day). Fasting was found to be safe and feasible for the cancer patients. There was also some preliminary evidence of reduced DNA-damage evident in host leukocytes after chemotherapy exposure for subjects who fasted for 72 h compared to 24 h in this study [20].

Our results confirm the feasibility and tolerability of STF accompanying chemotherapy and extend on these findings by indicating a potential beneficial effect on QOL, fatigue and well-being during cancer treatment. As QOL is an increasingly appreciated treatment outcome the present results appear to be of clinical relevance.

We did not find any evidence of malnutrition. Of note, the safety of fasting prior to chemotherapy can only be extrapolated to the selected population of oncology patients, as we excluded those with recent weight loss or a BMI < 19.0 kg/m^2^.

Our study has several limitations. The most obvious limitation is the small sample size within the context of an explorative pilot study, which limits the power of the study and precludes firm statistical conclusions. The second limitation is the cross-over design with the known bias of carry-over effects. However, we chose this design on the background of the defined small sample size and the respective likelihood of heterogeneous groups ruling out sound comparison of chemotherapy cycles with common randomization. Another limitation is due to the positive reputation of fasting cures in Germany. As such, participants in the study could be predisposed towards fasting, thereby inducing a non-specific effect.. This could only be addressed with a double-blind randomized study, which, however, is not feasible.

We aimed to include more patients with ovarian cancer, however, as these patients were frequently weakened by surgery, had more frequently experienced previous weight loss and were more often included in other clinical trials, the options for study recruitment were limited. The heterogeneity of the studied population and its treatments is a limitation for interpreting the results, however it reflects the current complexity of applied chemotherapies and reflects the external validity of the results. Finally, there is a possibility of underreporting and non-compliance with fasting, despite our encouragement to all study subjects to honestly disclose all food and beverages consumed during the fasting periods and the monitoring by the nutritionists. Yet, as fasting is widely established and positively reputed in Germany and all participants received detailed information before study inclusion about the fasting scheme we believe that the reported good compliance reflects the true compliance with the fasting protocol.

Of note, the beneficial effect of fasting on quality of life was more pronounced group in A compared to group B. There are several possible explanations for this differential effect. First, despite randomization Group A showed statistically nonsignificant but clinically relevant greater impaired quality of life than group B at baseline. This may have influenced the treatment response to fasting. Second, it could be that fasting is more effective if it prevents negative effects before they happen rather than after they occur. Third, patients in group A could have experienced more pronounced non-specific effects as they fasted first. In contrast, patients in group B might have decreased their negative expectation of the adverse effects of chemotherapy in the first cycles thereby reducing nonspecific effects of the following fasting intervention.

## Conclusion

Our study shows that STF during chemotherapy is feasible and has beneficial effects on QOL, well-being and fatigue. Larger randomized trials with confirmatory study design are warranted to further evaluate this innovative treatment approach.

## Abbreviations

DSR: Differential stress resistance
MID: MINIMALLY Important Difference
QOL: Quality-of-life
STF: Short-term fasting
